# A Cross-Sectional Study of Evening Hyperphagia and Nocturnal Ingestion: Core Constituents of Night Eating Syndrome with Different Background Factors

**DOI:** 10.3390/nu13114179

**Published:** 2021-11-22

**Authors:** Kentaro Matsui, Yoko Komada, Isa Okajima, Yoshikazu Takaesu, Kenichi Kuriyama, Yuichi Inoue

**Affiliations:** 1 Department of Laboratory Medicine, National Center Hospital, National Center of Neurology and Psychiatry, Tokyo 1878551, Japan; matsui.kentaro@ncnp.go.jp; 2 Department of Sleep-Wake Disorders, National Institute of Mental Health, National Center of Neurology and Psychiatry, Tokyo 1878553, Japan; kenichik@ncnp.go.jp; 3 Japan Somnology Center, Neuropsychiatric Research Institute, Tokyo 1510053, Japan; 4 Liberal Arts, Meiji Pharmaceutical University, Tokyo 2048588, Japan; yoko.komada@gmail.com; 5 Department of Psychological Counseling, Faculty of Humanities, Tokyo Kasei University, Tokyo 1738602, Japan; okajima-i@tokyo-kasei.ac.jp; 6 Department of Neuropsychiatry, Graduate School of Medicine, University of the Ryukyus, Okinawa 9030215, Japan; takaesuy@med.u-ryukyu.ac.jp; 7 Department of Somnology, Tokyo Medical University, Tokyo 1608402, Japan

**Keywords:** night eating syndrome, nocturnal eating, depression, anxiety, insomnia, distress, eating disorder

## Abstract

This web-based cross-sectional survey aimed to elucidate the differences between the two core symptoms of night eating syndrome (NES): evening hyperphagia and nocturnal ingestion in the general Japanese population aged 16–79 years. Participants who consumed at least 25% of daily calories after dinner were defined as having evening hyperphagia. Those who consumed food after sleep initiation at least twice a week were determined to have nocturnal ingestion. Of the 8348 participants, 119 (1.5%) were categorized in the evening hyperphagia group, 208 (2.6%) in the nocturnal ingestion group, and 8024 in the non-NES group. Participants with evening hyperphagia and nocturnal ingestion had significantly higher anxiety scores (*p* < 0.05 and *p* < 0.001, respectively) and depression (*p* < 0.001 for both) than those without NES. Multiple logistic regression analysis revealed that evening hyperphagia was significantly and independently associated with higher body mass index, shorter sleep duration, later sleep-wake schedule, and higher insomnia score, while nocturnal ingestion was significantly and independently associated with younger age, smoking habit, living alone, earlier sleep-wake schedule, and higher insomnia score. Sleep duration and sleep-wake schedule characteristics in the two groups were opposite, suggesting differences in the sleep pathophysiology mechanisms.

## 1. Introduction

Night eating syndrome (NES) is characterized by excessive eating just before going to bed or after waking up at night, which can result in difficulty in controlling body weight [1,2]. NES was first described by Stunkard et al. [3] in 1955 as a disease with features of morning anorexia, evening hyperphagia, and insomnia. Subsequently, nocturnal ingestion (i.e., waking during the sleep period to eat) was recognized as a symptom of NES as well [4,5]. NES has been reported to be associated with depression, distress, and sleep disorders [1,2], and it is listed as one of the “other specified feeding or eating disorders” in the Diagnostic and Statistical Manual of Mental Disorders, 5th Edition [6]. In the proposed diagnostic criteria of NES by Allison et al. [7], which was developed based on a review of previous literature, evening hyperphagia was defined as “At least 25% of the food intake is consumed after the evening meal” and nocturnal ingestion as “At least two episodes of nocturnal eating per week”.

Nocturnal ingestion shares a common pathophysiology with sleep-related eating disorder (SRED) [8,9], which is a parasomnia characterized by nocturnal eating behavior associated with total amnesia or partial unawareness of eating or drinking episodes [10,11,12,13]. Recently, we conducted a large epidemiological study in a young population using the Munich Parasomnia Screening questionnaire, a screening tool for parasomnias [14,15]. The study indicated an association between the possibility of NES and factors including female sex, smoking, use of hypnotic medications, history of sleepwalking, delayed sleep-wake schedules, and poor sleep quality [16]. However, this study had a few limitations. First, it was limited to a population of young adults. Second, even more importantly, the study only identified individuals with nocturnal ingestion and not those with evening hyperphagia [16]. 

A recent video polysomnography study showed that participants with nocturnal ingestion were younger and less obese than those with evening hyperphagia [17]. Additionally, in terms of eating and subsequent falling asleep behaviors, the participants in the evening hyperphagia group had significantly longer eating episodes and sleep resumption times after eating offset than those in the nocturnal ingestion group [17]. However, no studies have examined the differences in the psychological burden and lifestyle-related factors, including sleep variables, between evening hyperphagia and nocturnal ingestion. Therefore, in this study, we investigated the factors associated with evening hyperphagia and nocturnal ingestion in a large, stratified population, which covered a wide age range. Further, we compared the anxiety and depression scores between individuals with evening hyperphagia and those with nocturnal ingestion. Finally, we discuss the similarities and differences between the pathophysiology of evening hyperphagia and nocturnal ingestion with respect to sleep-wake schedules and social jet lag, after adjusting for the severity of insomnia symptoms.

## 2. Materials and Methods

### 2.1. Study Population and Outcomes

The present study was conducted as part of a comprehensive research project that investigated the sleep problems and daytime functioning in the general population [18]. This web-based cross-sectional questionnaire survey was conducted in April 2016. The participants were previously registered as research panel members of an established internet survey company and resided in all areas of Japan, stratified by age, sex, and region of residence. The study protocol was approved by the ethics committee of the Neuropsychiatric Research Institute, Tokyo, Japan. Informed consent was obtained from all participants via the survey website. 

A total of 10,000 participants responded to the questionnaire. Participants were excluded based on the following criteria: out of the age range of 16–79 years (*n* = 3), average sleep duration out of the mean ± 2 standard deviation (SD) range (402 ± 197 min, *n* = 265), sleep midpoint out of the mean ± 2 SD range (3:37 AM ± 3:39, *n* = 240), and shift workers (*n* = 1144). Finally, 8348 participants (83.5%) were included in the analyses. 

The following participant demographic information was obtained using the questionnaire: age, sex, body mass index (BMI), smoking status (“Do you currently smoke?”) and habitual alcohol intake (“Do you drink alcohol habitually?”), regular employment or school attendance (“Do you work or go to school regularly?”), frequency of working or going to school per week (“If yes, how many days a week do you work or go to school?”), family constitution (“Do you currently live alone or with your family?”), habitual exercise (“Do you exercise regularly: at least 30 min a day, 2 days a week, for 1 year?”) [19], and hypnotic medication use (“Do you currently take medicine to sleep?”). For evaluating the participants’ sleep habits, the following questions were included: weekday or days off bedtime (“What time do you usually go to bed on weekdays or days off?”), sleep onset latency (“How long does it usually take you to fall asleep on weekdays or days off?”), and wake-up time (“What time do you usually get up on weekdays or days off?”). The Japanese version of the Insomnia Severity Index (ISI) for assessing insomnia severity [20,21] and the Japanese version of the Hospital Anxiety and Depression Scale (HADS) for determining the severity of depression and anxiety [22,23,24] {Fulda, 2008 #117} were also included in the questionnaire.

The questionnaire also included four questions on the participants’ eating habits: frequency of eating dinner per week, time of last meal, frequency of nighttime food intake (“How many days per week do you eat anything between dinner and the next morning, such as a night snack or light meal?”), and percentage of nighttime food calories from the total daily calories (“Considering your total calories for the day as 100%, how many calories do you take after dinner up to the next morning?”). The questionnaire was used to identify the participants who met the two main items of the proposed diagnostic criteria of NES by Allison et al. [7]. Based on the A1 criterion (evening hyperphagia) of “At least 25% of the daily food is consumed after the evening meal,” participants who had dinner as well as snacked between dinner and the next morning and consumed at least 25% of their daily calories between dinner and bedtime every day were identified as having met the A1 criterion and defined as having evening hyperphagia. They included some participants who also met the A2 criterion [17]. According to the A2 criterion (nocturnal ingestion) of “At least two eating episodes per week occur upon awakening during the night,” participants whose last episodes of eating/drinking were later than their sleep onset time at least twice a week (both workdays and days off) were identified. The identified participants who only fulfilled the A2 criterion were defined as having nocturnal ingestion [17]. 

For the sleep variables, the sleep onset time was first calculated using bedtime and sleep onset latency. Next, the difference between sleep onset time and wake-up time was defined as sleep duration, while the midpoint between the sleep onset and wake-up times was defined as the sleep midpoint. Both parameters were assessed on weekdays and days off. The average sleep time was calculated using the following formula: ((sleep duration on weekdays × x + (sleep duration on days off × {7 − x}))/7, where x is the number of working days or school days in the week. Similarly, the average sleep midpoint was calculated using the following formula: ((sleep midpoint on weekdays × x) + (sleep midpoint on days off × {7 − x}))/7. Additionally, the absolute difference in sleep midpoint between weekdays and days off was calculated and defined as the absolute social jet lag [25].

### 2.2. Statistical Analysis

The Chi-square test (categorical variables) or Mann-Whitney’s U test and Dunn’s multiple comparison test (continuous variables) were performed for comparing the descriptive categories, including the demographic and sleep hygiene variables, ISI scores, and HADS anxiety and depression scores, among the three groups (i.e., non-NES, evening hyperphagia, and nocturnal ingestion groups). To investigate the factors associated with evening hyperphagia and nocturnal ingestion, logistic regression analysis was conducted for age (categorized; see below), sex, BMI (categorized; see below), current smoker (yes/no), habitual alcohol intake (yes/no), regular employment or school attendance (yes/no), living alone (yes/no), habitual exercise (yes/no), hypnotic medication use (yes/no), average sleep duration (categorized; see below), sleep-wake schedule (earlier/normal/later; earlier and later phases were defined by the first and third quartiles of the average sleep midpoint: earlier than 2:35 AM and later than 4:02 AM, respectively), absolute social jet lag (categorized; see below), and ISI score (categorized; see below). The aforementioned factors were categorized as follows: (1) age: 16–39, 40–59, and 60–79 years; (2) BMI: <25 and ≥25 kg/m^2^ [26]; (3) average sleep duration: <6 and ≥6 h (based on the cutoff for the first quartile: 357 min); (4) absolute social jet lag: <1, 1–2, and ≥2 h [18]; (5) ISI score: <8, 8–14, and 15–28 points [20,21]. Initially, all variables were examined using univariate models. The variables that showed significant correlation in the univariate analysis underwent multivariate logistic regression analysis for determining the main correlates while controlling for confounding factors. SPSS statistics version 22 (SPSS Japan, Inc., Tokyo, Japan) was used to conduct all analyses. Statistical significance was set at *p* < 0.05.

## 3. Results

Among the 8348 participants, 119 (1.5%) were categorized in the evening hyperphagia group (including four participants who met both the A1 and A2 criteria), 208 (2.6%) in the nocturnal ingestion group, and the remaining 8024 in the non-NES group. The median (range) for the continuous variables was as follows: age, 50 (16–79) years; BMI, 21.8 (12.0–49.1) kg/m^2^; average sleep duration, 400 (206–599) min; sleep midpoint, 3:17 (0:00–7:15) AM; absolute social jet lag, 0 (0–398) min; ISI score, 4 (0–28) points; HADS anxiety score, 4 (0–21) points, and HADS depression score, 8 (0–21) points. Descriptive information on the categorical variables of the participants is presented in Table 1.

### Descriptive and Clinical Variables

**Figure 1:**
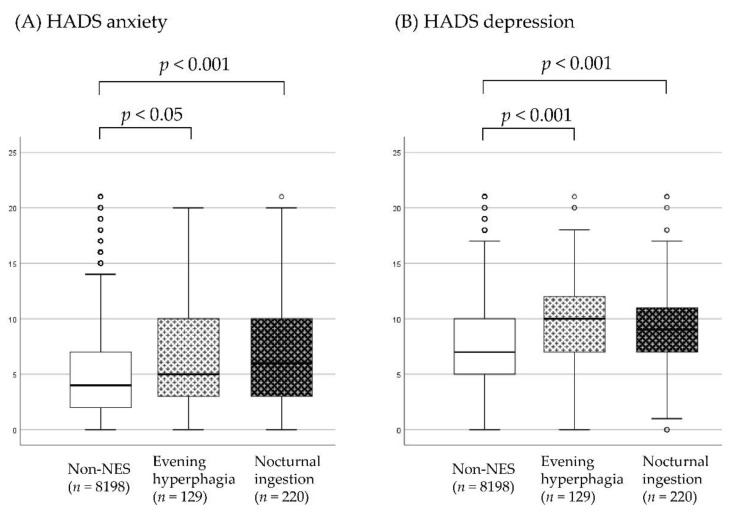
Box-and-whisker plot showing the Hospital Anxiety and Depression Scale (HADS) scores of the non-night eating syndrome (non-NES), evening hyperphagia, and nocturnal ingestion groups. HADs anxiety and depression scores are shown in (**A**,**B**), respectively. Comparisons were made using the Mann-Whitney U test.

The median (interquartile range (IQR)) scores of HADS anxiety for the non-NES, evening hyperphagia, and nocturnal ingestion groups were 4 (2–7), 5 (3–10), and 6 (3–10), respectively. The HADS anxiety score was significantly different among the three groups, H (2) = 38.39, *p* < 0.001, η^2^ = 0.0046. Pairwise comparisons showed higher anxiety levels in the evening hyperphagia (*p* < 0.05, Cohen’s *d* = 0.30) and nocturnal ingestion groups than in the non-NES group (*p* < 0.001, Cohen’s *d* = 0.43). The anxiety levels were not significantly different between the participants with evening hyperphagia and those with nocturnal ingestion (*p* = 0.374, Cohen’s *d* = 0.11). The median (IQR) scores of HADS depression for the non-NES, evening hyperphagia, and nocturnal ingestion groups were 7 (5–10), 10 (7–12), and 9 (7–11), respectively. Participants with evening hyperphagia and nocturnal ingestion had significantly higher depression scores than the non-NES participants (*p* < 0.001 for both). The HADS depression score was significantly different among the three groups, H (2) = 63.25, *p* < 0.001, η^2^ = 0.0076. Pairwise comparisons showed higher depression levels in the evening hyperphagia (*p* < 0.001, Cohen’s *d* = 0.53) and nocturnal ingestion groups than in the non-NES group (*p* < 0.001, Cohen’s *d* = 0.38). The depression levels were not significantly different between the participants with evening hyperphagia and those with nocturnal ingestion (*p* = 0.739, Cohen’s *d* = 0.15) (Figure 1). 

In the evening hyperphagia group, seven items were significantly associated in the univariate logistic regression analysis: age category of 60–79 years (odds ratio (OR) = 0.541, 95% confidence interval (CI): 0.330–0.887, *p* < 0.05), BMI of ≥ 25 kg/m^2^ (OR = 1.639, 95% CI: 1.089–2.468, *p* < 0.05), living alone (OR = 1.730, 95% CI: 1.134–2.640, *p* < 0.05), average sleep duration of < 6 h (OR = 2.345, 95% CI: 1.628–3.378, *p* < 0.001), later sleep-wake schedule (OR = 2.723, 95% CI: 1.834–44.044, *p* < 0.001), ISI score of 8–14 points (OR = 1.613, 95% CI: 1.066–2.441, *p* < 0.05), and ISI score of 15–28 points (OR = 3.538, 95% CI: 2.104–5.949, *p* < 0.001). Furthermore, in the multiple logistic regression analysis, significant associations were found for the following four items: BMI of ≥ 25 kg/m^2^ (OR = 1.525, 95% CI: 1.005–2.313, *p* < 0.05), average sleep duration of < 6 h (OR = 1.687, 95% CI: 1.152–2.472, *p* < 0.01), later sleep-wake schedule (OR = 2.196, 95% CI: 1.450–3.326, *p* < 0.001), and ISI score of 15–28 points (OR = 2.653, 95% CI: 1.558–4.515, *p* < 0.001) (Table 2). 

In the nocturnal ingestion group, nine items were significantly associated in the univariate logistic regression analysis: age category of 40–59 years (OR = 0.622, 95% CI: 0.453–0.855, *p* < 0.01), age category of 60–79 years (OR = 0.407, 95% CI: 0.285–0.581, *p* < 0.001), current smoker (OR = 1.544, 95% CI: 1.129–2.112, *p* < 0.01), living alone (OR = 1.618, 95% CI: 1.166–2.247, *p* < 0.01), hypnotic medication use (OR = 1.742, 95% CI: 1.137–2.667, *p* < 0.05), earlier sleep-wake schedule (OR = 1.804, 95% CI: 1.321–2.463, *p* < 0.001), absolute value of social jet lag of ≥2 h (OR = 1.694, 95% CI: 1.053–2.725, *p* < 0.05), ISI score of 8–14 points (OR = 1.705, 95% CI: 1.253–2.322, *p* < 0.001), and ISI score of 15–28 points (OR = 2.978, 95% CI: 1.949–4.548, *p* < 0.001). Furthermore, in the multiple logistic regression analysis, significant associations were found for the following seven items: age category of 40–59 years (OR = 0.532, 95% CI: 0.382–0.741, *p* < 0.001), age category of 60–79 years (OR = 0.355, 95% CI: 0.241–0.523, *p* < 0.001), current smoker (OR = 1.558, 95% CI: 1.129–2.152, *p* < 0.01), living alone (OR = 1.569, 95% CI: 1.121–2.196, *p* < 0.01), earlier sleep-wake schedule (OR = 2.153, 95% CI: 1.562–2.967, *p* < 0.001), ISI score of 8–14 points (OR = 1.577, 95% CI: 1.149–2.165, *p* < 0.01), and ISI score of 15–28 points (OR = 2.507, 95% CI: 1.571–4.001, *p* < 0.001) (Table 3).

## 4. Discussion

This is the first study to examine the prevalence of evening hyperphagia and nocturnal ingestion as well as their association with descriptive background variables, including sleep habits, in the general population across a wide age range. Here, 4.1% of the participants were suspected to have NES, out of which approximately two-thirds were categorized as having nocturnal ingestion. This prevalence was higher than that previously reported in the general population [27] but lower than that reported in individuals with obesity [28]. In the present study, both evening hyperphagia and nocturnal ingestion showed a significant association with insomnia severity. This result supports the original definition of NES by Stunkard et al. [3] and is consistent with the results of previous literature that showed an association between insomnia and NES, which was based on the definition including both evening hyperphagia and nocturnal ingestion [9,29]. This finding is also in line with the results of previous studies that indicated an association between insomnia and binge eating [30,31], suggesting a close link between insomnia and eating behavior, regardless of the time of food ingestion.

In this study, evening hyperphagia was significantly associated with shorter habitual sleep duration, independent of insomnia symptoms. A previous study indicated increased appetite due to chronic sleep deprivation [32] as one of the possible causative factors of evening overeating. In contrast, nocturnal ingestion was associated with longer average sleep duration. This is a puzzling result, considering that SREDs, another subtype of nocturnal ingestion, are more frequent in individuals with shorter sleep duration [8]. However, sleep duration in this study was calculated as the difference between sleep onset time in the night and wake-up time in the morning, and there were no data on wake time after sleep onset. A large difference between the sleep onset time and wake-up time suggests that a prolongation of time in bed due to exacerbation of sleep maintenance insomnia might lead to the occurrence of nocturnal ingestion [33]. However, a direct relationship between habitual sleep duration and evening hyperphagia or nocturnal ingestion has not been well elucidated to date, and this issue needs to be examined in future studies. One more contrasting feature observed in this study was that evening hyperphagia and nocturnal ingestion were independently associated with a later sleep-wake schedule and an earlier sleep-wake schedule, respectively. Considering the circadian rhythm perspective, the pathology of NES has been potentially linked with a delay in feeding rhythms deviating from sleep-wake rhythms [34,35]. However, because of the reports of delayed sleep-wake rhythms in patients with NES [16,36], the relationship between feeding rhythms and sleep-wake rhythms remains controversial. The inconsistencies in the sleep-wake rhythm patterns among patients with NES in previous reports were possibly attributed to the distinct association of the sleep-wake schedules between the patients with evening hyperphagia and those with nocturnal ingestion. 

Among the three groups, the anxiety and depression scores were significantly higher in the evening hyperphagia and nocturnal ingestion groups than in the non-NES group. The effect sizes of Cohen’s *d* were small to moderate in both comparisons, indicating that these results could be applied in daily clinical practice. Although many reports suggest an association between NES and depression [1,37,38,39,40,41,42,43,44,45,46,47,48,49,50,51], only a few reports show an association between NES and anxiety [51,52]. In the present study, both evening hyperphagia and nocturnal ingestion had similar levels of association with anxiety and depression, possibly due to the underlying covariation of anxiety and depression [53]. Administration of selective serotonin reuptake inhibitors (SSRIs) has been indicated as an effective treatment for NES [54,55,56,57]. They alleviate the eating symptoms, possibly via reduction in anxiety and depression. However, how SSRIs affect nighttime appetite and eating behavior remains unclear. Future studies should conduct detailed examinations of the relationship between the improvement of nighttime eating frequency/severity and anxiety/depression.

Consistent with the results of our previous study [16], hypnotic medication use was more common in the evening hyperphagia and nocturnal ingestion groups than in the non-NES group. However, it was not independently associated with the evening hyperphagia or nocturnal ingestion groups. The increased use of hypnotic medication might be a resultant phenomenon of the relation between evening hyperphagia and nocturnal ingestion with insomnia. However, simultaneously, it should be noted that the type and dosage of hypnotic medications were not examined in this study, and the impacts of hypnotics usage might be underestimated. Additionally, since the nocturnal ingestion group in the present study was quite young, this group might have exhibited characteristics that were common with SREDs, which are non-rapid eye movement parasomnias that commonly occur in the younger populations [13] and can be induced by hypnotic GABA_A_ agonist medication [58,59,60,61,62,63]. Furthermore, another area of interest for future research would be the aspect of nocturnal eating with amnesia, which was not investigated in this study.

The relationship between NES and body weight remains controversial. Although NES is common in patients with obesity, many patients with NES do not present with obesity [64]. In this study, all three groups did not show a significant difference in BMI, suggesting that NES morbidity is not associated with weight gain in the general population. However, it was only evening hyperphagia and not nocturnal ingestion, that showed an independent and significant association with a higher BMI. This finding suggests that individuals with high BMI are more prone to developing evening hyperphagia, which is partly in line with a report showing that obesity is less common in individuals with nocturnal ingestion than in those with evening hyperphagia [17]. In individuals with obesity, the comorbidity of NES interferes with weight loss [65,66], but this phenomenon might be caused by evening hyperphagia and not nocturnal ingestion. Future studies should clarify the differences in the weight loss interference between evening hyperphagia and nocturnal ingestion. 

Considering the other lifestyle-related factors, the percentage of smokers was higher in the evening hyperphagia and nocturnal ingestion groups, where smoking was independently associated with nocturnal ingestion. This was consistent with the results of our previous study [16], a report showing a high rate of nicotine dependence in patients with NES [44], and a retrospective study on NES/SRED outpatients in which nearly 40% were smokers [58]. A smoking habit is more prevalent in patients with eating disorders [67], especially in those with bulimia nervosa and binge eating disorder rather than in those with anorexia nervosa [68,69,70]. Additionally, addictive traits in smokers [71] might be associated with nocturnal ingestion. As for the other sociodemographic factors, living alone status was frequent in the evening hyperphagia and nocturnal ingestion groups, where it was independently associated with nocturnal ingestion. This may be because individuals who live alone are more likely to have disrupted lifestyles, including eating habits [72,73]. Furthermore, social jet lag, which might lead to insulin resistance via circadian disruption [74], was not significantly associated with either evening hyperphagia or nocturnal ingestion. This suggests that social jet lag, which includes weekday sleep deficits and delayed weekend sleep schedules, does not contribute to NES pathology on its own. 

There are some limitations in the present study. First, the detection of NES relied only on the self-administered questionnaires and was not determined by clinical interviews. Moreover, the identification of evening hyperphagia according to the caloric intake after dinner was based on self-reports, while the definition of nocturnal ingestion, which was based on the last time of food consumption, time to go to bed, latency to fall asleep on weekdays and holidays, and frequency of eating after bedtime, was not assessed directly. In addition, the content and amount of foods ingested were not clear; thus, the possible negative effects of the diet could not be fully investigated. To address these limitations, a detailed assessment of the number of consumed calories and day-to-day variation in eating behavior should be conducted. Additionally, lack of data on wake time after sleep onset was another methodological limitation. Second, this study was conducted as an internet survey. Studies suggest that internet users tend to have shorter sleep duration and delayed sleep-wake rhythms [75,76], which might have led to a sampling bias. Additionally, this was a cross-sectional study and, therefore, a causal relationship cannot be identified between the identified factors and evening hyperphagia/nocturnal ingestion. Third, smoking and drinking habits in this study were determined by responses of “yes” or “no”; therefore, our study did not reflect the amount or timing of exposure to nicotine and alcohol. Moreover, this study did not have information on comorbidities and concomitant medications. Although comorbid sleep disorders, such as narcolepsy, obstructive sleep apnea, and periodic limb movement disorder are associated with NES [4,10], and comorbidities of certain endocrine disorders and the use of psychotropic drugs may increase appetite [77,78], information on these issues was lacking.

## 5. Conclusions

In the present study, we examined factors associated with the two main constituent symptoms of NES, evening hyperphagia and nocturnal ingestion. Our findings showed that participants with either of these core symptoms exhibited higher anxiety and depression scores than those without NES. Evening hyperphagia was associated with shorter habitual sleep duration and delayed sleep-wake schedules, while nocturnal ingestion was linked to longer habitual sleep duration and earlier sleep-wake schedules, suggesting different pathophysiological backgrounds between the two symptoms. These findings are important for devising therapeutic interventions for NES, such as the improvement of sleep-wake rhythms and sleep duration for severe evening hyperphagia. Moreover, the relationship between NES and sleep habits should be examined in clinical populations to confirm the significance of sleep schedule in the mechanism of NES. Furthermore, future longitudinal studies are warranted to clarify the impact of NES on physical and mental health.

## Figures and Tables

**Table 1:** Demographic, sleep schedules, and clinical data of the participants (*n* = 8348).

	Non-NES Group (*n* = 8021)	Evening Hyperphagia Group (*n* = 119)	Nocturnal Ingestion Group (*n* = 208)	*p*
Age, median (range), years	50 (16–79)	47 (21–72) ^3^	41.5 (19–79) ^1^	<0.001
Female, *n* (%)	4024 (50.2)	66 (55.5)	92 (44.2)	n.s.
BMI, median (range), kg/m^2^	21.8 (12.0–49.1)	21.5 (15.1–32)	21.4 (13.3–34.6)	n.s.
Current smoker, *n* (%)	1506 (18.8)	31 (26.1) ^5^	55 (26.4) ^5^	<0.01
Habitual alcohol intake, *n* (%)	3515 (43.8)	56 (47.1)	101 (48.6)	n.s.
Regular employment or school attendance, *n* (%)	4720 (58.8)	70 (58.8)	128 (61.5)	n.s.
Living alone, *n* (%)	1244 (15.5)	29 (24.4) ^4^	48 (23.1) ^4^	<0.001
Regular physical activity, *n* (%)	2748 (34.3)	32 (26.9)	81 (38.9)	n.s.
Hypnotic medication use, *n* (%)	579 (7.2)	13 (10.9) ^6^	25 (12.0) ^6^	<0.05
Average sleep duration, median (range), min	400 (206–599)	375 (225–595) ^3^	418.3 (220–595) ^1^	<0.001
Sleep midpoint, median (range), h: min	3:17 (0:00–7:15)	3:59 (0:18–7:15) ^1^	3:03 (0:15–6:49) ^2^	<0.001
Absolute social jet lag, median (range), h	0 (0–398)	0 (0–390)	15 (0–360) ^3^	<0.05
ISI score, median (range), points	4 (0–28)	6 (0–28) ^1^	7 (0–27) ^1^	<0.001
HADS anxiety score, median (range), points	4 (0–21)	5 (0–20) ^1^	6 (0–21) ^1^	<0.001
HADS depression score, median (range), points	7 (0–21)	10 (0–21) ^1^	9 (0–21) ^1^	<0.001
Age, median (range), years	50 (16–79)	47 (21–72) ^3^	41.5 (19–79) ^1^	<0.001

^1^ *p* < 0.001 compared to the non-NES group. ^2^ *p* < 0.01 compared to the non-NES group. ^3^ *p* < 0.05 compared to the non-NES group. ^4^ *p* < 0.001 defined by adjusted rest errors. ^5^ *p* < 0.01 defined by adjusted rest errors. ^6^ *p* < 0.05 defined by adjusted rest errors. The Chi-square test was conducted for categorical variables, while Mann-Whitney’s U test and Dunn’s multiple comparison test were conducted for continuous variables. Non-NES, non-night eating syndrome; n.s., not significant; BMI, body mass index; ISI, Insomnia Severity Index; HADS, hospital anxiety and depression scale.

**Table 2:** Factors associated with evening hyperphagia (*n* = 119).

Predictor	*n*	Univariate Relative Risk (95% CI) ^1^	*p*	Multivariate Relative Risk (95% CI) ^1^	*p*
Age (years)					
16–39	2544				
40–59	2843		n.s.		n.s.
60–79	2961	0.541 (0.330–0.887)	<0.05		n.s.
Sex					
Male	4166				
Female	4182		n.s.		n.s.
BMI (kg/m^2^)					
<25	6808				
≥25	1540	1.639 (1.089–2.468)	<0.05	1.525 (1.005–2.313)	<0.05
Current smoker					
No	6756				
Yes	1592		n.s.		n.s.
Habitual alcohol intake					
No	4676				
Yes	3672		n.s.		n.s.
Regular employment or school attendance					
No	3430				
Yes	4918		n.s.		n.s.
Living alone					
No	7027				
Yes	1321	1.730 (1.134–2.640)	<0.05		n.s.
Regular physical activity					
No	5487				
Yes	2861		n.s.		n.s.
Hypnotic medication use					
No	7731				
Yes	617		n.s.		n.s.
Average sleep duration (h)					
≥6	6196				
<6	2152	2.345 (1.628–3.378)	<0.001	1.687 (1.152–2.472)	< 0.01
Sleep-wake schedule					
Normal	4217				
Earlier ^2^	2053		n.s.		n.s.
Later ^3^	2078	2.723 (1.834–4.044)	<0.001	2.196 (1.450–3.326)	<0.001
Absolute social jet lag (h)					
<1	6500				
1–2	1335		n.s.		n.s.
≥2	513	1.852 (1.028–3.336)	< 0.05		n.s.
ISI score (points) ^4^					
0–7	5875				
8–14	1974	1.613 (1.066–2.441)	< 0.05		n.s.
15–28	499	3.538 (2.104–5.949)	< 0.001	2.653 (1.558–4.515)	<0.001

^1^ Relative risks approximated to odds ratios. ^2^ Earlier phase was defined using the first quartile of the average sleep midpoint: earlier than 2:35 AM. ^3^ Later phase was defined using the third quartile of the average sleep midpoint: later than 4:02 AM. ^4^ Interpreted as follows: absence of insomnia (0–7), subthreshold insomnia (8–14), and moderate to severe insomnia (15–28). CI, confidence interval; BMI, body mass index; ISI, Insomnia Severity Index; n.s., not significant.

**Table 3:** Factors associated with nocturnal ingestion (*n* = 208).

Predictor	*n*	Univariate Relative Risk (95% CI) ^1^	*p*	Multivariate Relative Risk (95% CI) ^1^	*p*
Age (years)					
16–39	2544				
40–59	2843	0.622 (0.453–0.855)	<0.01	0.532 (0.382–0.741)	<0.001
60–79	2961	0.407 (0.285–0.581)	<0.001	0.355 (0.241–0.523)	<0.001
Sex					
Male	4166				
Female	4182		n.s.		n.s.
BMI (kg/m^2^)					
<25	6808				
≥25	1540		n.s.		n.s.
Current smoker					
No	6756				
Yes	1592	1.544 (1.129–2.112)	< 0.01	1.558 (1.129–2.152)	<0.01
Habitual alcohol intake					
No	4676				
Yes	3672		n.s.		n.s.
Regular employment or school attendance					
No	3430				
Yes	4918		n.s.		n.s.
Living alone					
No	7027				
Yes	1321	1.618 (1.166–2.247)	<0.01	1.569 (1.121–2.196)	<0.01
Regular physical activity					
No	5487				
Yes	2861		n.s.		n.s.
Hypnotic medication use					
No	7731				
Yes	617	1.742 (1.137–2.667)	< 0.05		n.s.
Average sleep duration (h)					
≥6	6196				
<6	2152		n.s.		n.s.
Sleep-wake schedule					
Normal	4217				
Earlier ^2^	2053	1.804 (1.321–2.463)	<0.001	2.153 (1.562–2.967)	<0.001
Later ^3^	2078		n.s.		n.s.
Absolute social jet lag (h)					
<1	6500				
1–2	1335		n.s.		n.s.
≥2	513	1.694 (1.053–2.725)	<0.05		n.s.
ISI score (points) ^4^					
0–7	5875				
8–14	1974	1.705 (1.253–2.322)	<0.001	1.577 (1.149–2.165)	<0.01
15–28	499	2.978 (1.949–4.548)	<0.001	2.507 (1.571–4.001)	<0.001

^1^ Relative risks approximated to odds ratios. ^2^ Earlier phase was defined using the first quartile of the average sleep midpoint: earlier than 2:35 AM. ^3^ Later phase was defined using the third quartile of the average sleep midpoint later than 4:02 AM. ^4^ Interpreted as follows: absence of insomnia (0–7), subthreshold insomnia (8–14), and moderate to severe insomnia (15–28). CI, confidence interval; BMI, body mass index; ISI, Insomnia Severity Index; n.s., not significant.

## Data Availability

The data will be shared on reasonable request to the corresponding author.
